# Introduction of transitional curriculum (TC) for the first-year undergraduate in Ayurveda, a welcome move by the Ministry of Ayush

**DOI:** 10.1016/j.jaim.2022.100551

**Published:** 2022-03-04

**Authors:** Gaurav Soni, Neelam

**Affiliations:** Department of Rachana Sharir, North Eastern Institute of Ayurveda and Homoeopathy (NEIAH), India; Department of Dravya Guna, North Eastern Institute of Ayurveda and Homoeopathy (NEIAH), India

Editor,

Recently Central Council of Indian Medicine (CCIM), on behalf of the Ministry of Ayush, New Delhi; introduced a fifteen-day; ninety hourly, transitional curriculum (TC) for first-year of academic session 2020–21, for Ayurveda, Siddha, and Unani (ASU) streams. This curriculum was to be implemented by all ASU undergraduates colleges as part of their induction program making it a common practice all over the country. This sort of introductory program was much needed long since, to fill the gap of appreciation between the NEET aspirant and a newly admitted ASU undergraduate students.

The hierarchal arrangement of our education system conveys many transitional junctures in the student's life especially at the entrance into professional colleges [1]. Medical aspirants, have to initially clear the National Eligibility cum Entrance Test (NEET) and as per their performance in the entrance test, get enrolled in Ayush institution. In this transitory phase, students who come from diverse backgrounds, deliberations, and preparations; need the support from the organizations to experience a sense of belonging, bonding, awareness about their responsibilities and rights. A well-designed induction program helps both the students and faculties to set up a productive learning experience [2].

Induction programs are already customary for new entrants to colleges affiliated with the University Grants Commission (UGC), All India Council for Technical Education (AICTE) & Medical Council of India (MCI) [3]. Being professionally associated with an Ayurveda institute, the author would like to elaborate on, analyze and recognize the effectiveness of various parameters of prescribed TC, in the life of a first-year Bachelor of Ayurvedic Medicine and Surgery (BAMS) student.

Ancient texts have described the *Shisyopanayaneeya Sanskara* (Orientation and Induction ritual ceremony for newly admitted disciples) as an induction program before starting the formal studies. Sage Susruta, has described the S*hisyopanayaneeya* process and its significance at the very beginning of his compendium. Under this orientation program, students were enlightened with the ethics of the medical profession, rules of *Gurukul* (an ancient form of residential schools), the endeavor of education, and the character of good physicians. Additionally a great social reform used to be undertaken by providing all of them a common prefix i.e. *Vaidya* (Physician) after which caste/economic status didn't matter; that imparted social equality [4].

Recently the Ministry of Ayush has introduced a TC based on SAGE (Socializing, Associating, Governing, and Experiencing) aspects of the induction program is introduced. Every SAGE criterion has a definite goal and they are as follows - "Socializing" is for enabling the students to be comfortable; "Associating" is for getting the feeling of inclusion/belonging and also expunging the anxiety; "Governing" provides information about instituitional rules and regulations as well as the program, and "Experiencing" is for imparting students the preparatory view of the subject matter and developing basic skills. The intended objectives of the SAGE can be achieved through different activities including mentoring, guidence by prominent personalities of the Ayurveda through lectures, visits to campus/departments, recriational activities, cultural events, visits to the local community, etc. [5] The induction program by the Ministry of Ayush complies with all the above criterias making it one of the complete set of activities to fulfill all expectations of the new entrant in the institute; that are summarized in Table 1.

**Table 1:** Summary of activities under TC; along with their SAGE categories and; scheduled day and allocated time.

SNO	Activity	SAGE criterion	Day	Time Allocated (in hours)
1	Inaugural Function; Interaction with Parents & Students	S	1	4
2	The uniqueness of Ayurveda/Siddha/Sowa Rigpa/Unani (ASSU) compared to other systems of Medicine^a^	E	1	1
3	Language (*Vadatu Sanskritam*)^a^	E	1–12	14
4	History, Philosophy, and concepts of ASU- Introduction by an eminent person.^a^	E	2–4	3
5	Introduction about Modern Medicine and other systems of Medicine	E	2	1
6	Role of ASU in Public Health and Primary Health Centre^a^	E	2	1
7	Know your institute and department visit	A	2–4	3
8	Motivational Lecture including by eminent person of ASU^b^	E	2,12	3
9	Recent advances in ASU^a^	E	3–4	2
10	Know Regulatory bodies & Statutory bodies and major AYUSH institutions	G	3–4	2
11	Lecture by an eminent person of ASU^b^	S	3-4; 8-9	4
12	Clinical exposure	E	5–7	3
13	Role and impact of Physician in society^b^	S	5–6	2
14	Basics of *Prakriti Pareeksha*^a^	E	5–6	2
15	Skills – Basic Life Support (BLS) and First Aid^a^	E	5–6	4
16	*Vrikshaayurveda* and *Mrigayurveda*^a^	E	7	1
17	Professional Medical Ethics	G	7	1
18	Computer skills^a^	E	7,10	2
19	Games and sports grouping students and giving tasks	S/A	7–10	4
20	Communicative English^a^	E	8–10	3
21	Biodiversity and ASU^a^	E	8–9	2
22	Yoga and Meditation; and Relaxation Techniques	S/A	8–10	3
23	Know your syllabus, lectures by HoD	A	10	1
24	National Health Status, Goals, and Policies	E	11	1
25	Lecture by eminent Pharmaceutical industrialist/Researcher/Educationist etc^b^	E	11	1
26	Stress management and Capacity building to address medical challenges^b^	E	11	2
27	Videos related to AYUSH^a^	E	11	1
28	Discussion, debate, Tasks	S/A	12	1
29	Feedback by the students	S/A	12	1
30	Importance of Diet in ASU	E	13	2
31	Personal Health & Hygiene (*Dinacharya*)	E	13	1
32	Time Management^b^	E	13	1
33	Biomedical Waste Management	E	13	1
34	Understanding interpersonal relationships in a health care team^b^	A	14	1
35	Understanding the role of mentoring^b^	E	14	1
36	Comprehend the learning Pedagogy and the role in learning skills^a^	E	14	2
37	Understanding the process of group learning and group dynamics^b^	E	14	1
38	Understanding different methods of self-learning and collaborative learning^b^	E	14	1
39	Field visit to other institutions, eminent practitioners, AYUSH hospitals/dispensaries, Pharmaceuticals, and other institutions	A	15	6
**Total**				**90**

**Acronym SAGE (S-**Socializing; **A-** Associating; **G-** Governing; **E-** Experiencing).

a Academic & Skill Development Activities.

b Motivational/Mentoring Activity.

Among the different aspects of TC implemented, mentoring is the important part of the induction program (> 17% of scheduled time) that imparts confidence in students to work with the faculties. It also provides the joy of learning along with inculcating human values, such as sensitivity which are very important for a medical professional. The topics to be discussed under mentorship are aspirations, family expectations, quality of gratitude, time management and handling of peer pressure, prosperity, and importance of relationships. During discussions thoughts are shared among students and mentors that helps to develop a feeling of faithfulness among them; this also helps to develop self-confidence in the students and enable them to relate with faculties through both problems and solutions.

The next imperative step is introducing subject and language because *Ayurveda* philosophy is different from the contemporary era and classical literature is written in *Sanskrit*. TC has allocated > 40% of the time for introducing principles of *Ayurveda* along with basic learning skills. The lecture series comprising an introduction to *Ayurveda* philosophy, the role of ASU in the health care system, basic principles like *Prakriti Pareeksha, Vrikshaayurveda, Mrigayurveda*, etc; along with a basic introduction and practical utility of the *Sanskrit* language; computer knowledge, and communicative English; providing an adequate purview of the entire curriculum in a short span, which enables students to connect with their system of medicine.

The success of induction program is accessed through the feedback given by the students from time to time. It can be taken at end of the induction program or even at the mid or end of the semester/year. A similar seven days induction program took place in the Medical Education Unit of Osmania Medical College, in Hyderabad, Telangana for 190, first-year M.B.B.S. students of the 2014 batch, reported the induced skills to be useful and relevant in remarkable percentage [6]. A separate study held in Chennai at the Medical Education Unit, Sri Ramachandra Medical College, and Research Institute; also reported positive results of the induction program, which was conducted for 250 first-year undergraduate students [7].

This new introductory program is a game-changer for newly admitted Ayush undergraduates. Appropriate division of schedule makes it compatible as well as rewarding due to choice of topics and activities. Mentoring, motivation, subject introduction, along with local community visits make it a comprehensive set of activities for induction programs; that are helpful for new students to relinquish their anxiety and enjoy the new professional life with enthusiasm and ultimately becoming a responsible citizen of the country by serving humanity. In the end, regards to the Ministry of Ayush & their team; for introducing such a productive and efficient TC.

## Conflict of interest

None.

## Source of funding

None.
